# Sarcopenia, severe anxiety and increased C-reactive protein are associated with severe fatigue in patients with inflammatory bowel diseases

**DOI:** 10.1038/s41598-021-94685-5

**Published:** 2021-07-27

**Authors:** Laura Tasson, Fabiana Zingone, Brigida Barberio, Romina Valentini, Pamela Ballotta, Alexander C. Ford, Marco Scarpa, Imerio Angriman, Matteo Fassan, Edoardo Savarino

**Affiliations:** 1 Clinical Nutrition Unit, Department of Medicine-DIMED, University of Padova, Padua, Italy; 2 Gastroenterology Unit, Department of Surgery, Oncology and Gastroenterology, DISCOG, University of Padua, Via Giustiniani 2, 35121 Padova, Italy; 3 Leeds Gastroenterology Institute, St. James’s University Hospital, Leeds, UK; 4 Leeds Institute of Biomedical and Clinical Sciences, University of Leeds, Leeds, UK; 5 Clinica Chirurgica 1, Department of Surgery, Oncology and Gastroenterology, University of Padova, Padova, Italy; 6 Department of Medicine (DIMED), Surgical Pathology & Cytopathology Unit, University of Padua, Padua, Italy

**Keywords:** Gastroenterology, Medical research

## Abstract

Patients with inflammatory bowel disease (IBD) report fatigue more frequently than healthy population, but the precise mechanisms underlying its presence are unknown. This study aimed to evaluate the prevalence of fatigue in IBD and its relation with potential causative factors. A survey on fatigue, depression, anxiety, sleep disorders, and the presence of sarcopenia and malnutrition, was sent by email to 244 IBD outpatients of the Gastroenterology Unit of Academic Hospital of Padua**.** Demographics and clinical data, including the levels of fecal calprotectin (FC) and C-reactive protein (CRP), and current pharmacological treatments were obtained from patients’ medical records. Ninety-nine (40.5%) subjects answered the survey. Ninety-two (92.9%) patients reported fatigue, with sixty-six having mild to moderate fatigue and twenty-six severe fatigue. Multivariate analysis showed that abnormal values of CRP (OR 5.1), severe anxiety (OR 3.7) and sarcopenia (OR 4.4) were the factors independently associated with severe fatigue. Fatigue has a high prevalence in subject affected by IBD. Subjects with altered CRP, sarcopenia and severe anxiety appear more at risk of severe fatigue.

## Introduction

Inflammatory bowel diseases (IBD), including Crohn’s disease (CD) and ulcerative colitis (UC), are chronic inflammatory diseases affecting the gastrointestinal tract^1^. IBD is a lifelong condition without a cure, and because of the chronicity of symptoms and progression to disability, particularly in CD, patients with IBD may experience impaired quality of life, psychological illness, including anxiety and depression, sleep disturbance, and fatigue^2–6^. Moreover, patients affected by IBD are subjected to a major risk of malabsorption and reduction of food intake^7,8^. Finally, IBD patients tend to have a sedentary lifestyle, although physical activity is not contraindicated in this population^8^. All these factors contribute to an increased risk of developing protein energy malnutrition and sarcopenia^9^.

Fatigue is defined as an “overwhelming sense of tiredness, reduced energy levels and feeling of exhaustion that is not responsive to prolonged rest or sleep”^7,10^. In people affected by IBD, fatigue has been reported by up to 80% of patients with active disease and up to 54% of patients in remission^11^ twice more common than healthy controls^12^, and it is commonly defined as the most debilitating symptom during the period of disease inactivity^9,13^. Fatigue impacts negatively on every day occupations, on concentration during social interaction and causes the suspension of physical activity^14^. The self-imposed restrictions caused by fatigue elicit feelings of anger, resentment, self-pity and sadness, causing worsening of the quality of life. Causal factors are unknown because of the multifactorial nature of the symptom^13^. When measured with functional scales, fatigue is more prevalent in young women, those who are physically inactive, with a short history of disease, and who declare feeling stressed, depressed, or anxious^15–17^. Moreover, fatigue has been linked with a sub clinical pro-inflammatory state; it seems in fact that the complex formed by IL-6 and the IL-6 receptor, as well as TNFα, causes a break in the blood–brain barrier with effects on the central nervous system, provoking fatigue, anxiety, and depression^18^. Corticosteroids have been also implicated in the genesis of fatigue, while anti-TNF therapy seems to be protective^19^. Finally, the presence of micro- or macro-intestinal blood leaks, iron malabsorption, the suppression of erythropoiesis and the presence of pro-inflammatory cytokines have been associated with a high prevalence of anemia in IBD, partially correlated with the presence of fatigue^11^. However, to date, a clear causal relationship between fatigue and iron or other micronutrient deficiencies has not been established^9,11,20^.

An appealing hypothesis is that fatigue could be due to a reduction in lean mass, a condition caused by a combination of malabsorption, chronic inflammation and sedentary lifestyle^8,14,15^. Data supporting this theory come from the analysis of muscle strength and endurance indices in a sample of IBD patients, which showed a significant relation between asthenia and loss of muscle mass^21^.

This study aimed to examine the prevalence of fatigue in subject affected by IBD and its relationship with sarcopenia and other possible causative factors.

## Methods

### Study design

The present cross-sectional study was conducted at IBD Unit of Azienda Ospedaliera di Padova (Italy). Consecutively and prospectively, all IBD patients who visited our IBD Unit from February to July 2020 were asked to participate in this study. The study protocol conforms to the ethical guidelines of the 1975 Declaration of Helsinki as reflected in a priori approval by the institution’s human research committee of Padova University (protocol number 4197/AO/17). Informed consent was obtained from all individual participants included in the study.

A survey was conducted sending out an email to 244 IBD outpatients. Inclusion criteria were: age over 18, a histologically-confirmed diagnosis of CD or UC for a least 6 months, and a recent outpatient encounter with the need to perform biochemical assessment of disease activity. Exclusion criteria included the presence of known psychiatric disorders, history of alcohol or drug abuse, pregnancy, neoplasia or other systemic disorders potentially influencing the psychological status (i.e. connective tissue disorders, fibromyalgia, diabetes mellitus, autonomic or peripheral neuropathy, and myopathy), and the lack of consent. Demographics and clinical data, including the levels of fecal calprotectin (FC) C-reactive protein (CRP), and current pharmacological treatments were obtained from patients’ medical records. For the purpose of the study, active disease was defined by the presence of FC values ≥ 250mcg/g and/or a value of Harvey Bradshaw Index > 4 for patients with CD and a partial Mayo score > 1 for patients with UC^22,23^. Moreover, abnormal CRP was considered when higher than 0.1 mg/dL.

The survey consisted of different questionnaires to be filled after initial formal and written approval of participation into this study. The time required to complete the questionnaire was approximately 45 min. The domains and questionnaires used were:

- *Fatigue assessment.* To measure fatigue we used the IBD-F questionnaire, specifically developed for IBD patients^13,24,25^. The questionnaire is composed of two sections: the first examines the presence and the extent of fatigue, the second its impact on different aspects of daily living^26^. A score from 1 to 10 is suggestive of mild to moderate fatigue, a score from 11 to 20 of severe fatigue^25^.
- *Sarcopenia assessment.* Due to SARS-CoV-2 pandemic, we evaluated the presence of sarcopenia only through the SARC-F questionnaire, recommended by the European Consensus to elicit the declaration of sarcopenia’s signs and symptoms^27^. Indeed, there are five SARC-F components: strength, assistance with walking, rising from a chair, climbing stairs, and falls. The scores range from 0 to 10, with 0 to 2 points for each component. Preliminary studies have suggested that a score equal to or greater than 4 is predictive of sarcopenia and poor outcomes.
- *Depression and anxiety assessment.* Anxiety and depression were assessed via the Hospital Anxiety and Depression Scale, a questionnaire validated for epidemiological and clinical studies^28,29^, which has been demonstrated to be reliable when applied to IBD patients^19^. The questionnaire is formed of two scales, each one composed of seven items. Normal scores for anxiety or depression are defined by a score less than 8^30,31^. A subscore from 8 to 10 is defined as borderline abnormal, and a score from 11 to 21 as abnormal^32^.
- *Sleep disorders assessment.* Sleep quality has been evaluated through the Pittsburgh Sleep Quality Index, a questionnaire that examines seven different sleep components: subjective sleep quality, sleep latency, length, efficiency, sleep disturbances, drug use and daytime dysfunctions. The sum of each component gives a score from 0 to 21. A score of 5 or above suggests poor sleep quality^33^.
- *Quality of life assessment.* Quality of life was measured through the Inflammatory Bowel Disease Questionnaire Short Form^34^, specifically developed for testing subjects affected by these pathologies. It is widely validated, and has been demonstrated to be reliable and sensitive^35^. It is composed of ten questions, a subset of the full Inflammatory Bowel Disease Questionnaire, graded on a 7-point Likert scale from 1 (a very severe problem) to 7 (not a problem at all)^36^. A maximum of 70 indicates the best IBD related quality of life, a minimum of 10 indicates the worst^36^.
- *Malnutrition assessment.* Malnutrition was diagnosed according to the Global Leadership Initiative on Malnutrition criteria, namely in the presence of a weight loss greater than the 5% within the past 6 months or a BMI < 20 if the subject was aged less than 70 years old, or a BMI < 22 if the subject was 70 years old or older, and the concomitant presence of active inflammatory disease or an abnormal value of CRP^37^.

### Statistical analysis

Categorical variables were expressed as frequency and continuous variables as mean ± standard deviation (SD). We used univariate logistic regression models to assess whether demographic or IBD-related variables, HADS, IBDQ, PSQI, sarcopenia, or malnutrition were related to severe fatigue. Statistically significant variables in univariate analyses were then included in a multivariate regression model to identify, using the AIC stepwise method, independent risk factors for fatigue. A p-value < 0.05 was considered statistically significant. STATA 11 software was used to perform statistical analysis.

### Ethics committee approval

The study protocol conforms to the ethical guidelines of the 1975 Declaration of Helsinki as reflected in a priori approval by the institution’s human research committee of Padova University, Italy (protocol number 4197/AO/17).

### Informed consent

Informed consent was obtained from all individual participants included in the study.

## Results

### Characteristics of study participants

Ninety-nine out of 244 (40.5%) IBD outpatients followed at our IBD unit and enrolled in the study, answered the survey by email. Table 1 summarizes the main demographic and clinical characteristics of the study population. The mean age of subjects was 38.7 ± 13.9 years, there were 57.5% males and 54.5% patients affected by CD. Almost 50% had a duration of disease longer than 10 years. Among the entire population, 30% had clinically and/or biochemically active disease, 44% were taking biological therapies, and only 3% were on a corticosteroid.

**Table 1:** Demographical and clinical characteristics of the 99 IBD patients who answered the survey.

Features	IBD patients (n = 99)
**Age, mean ± sd**	38.7 ± 13.9
**Sex, n (%)**	
Male	57 (57.5%)
Female	42 (42.4%)
**Type of disease, n (%)**	
Crohn’s disease	54 (54.5%)
Ulcerative Colitis	45 (45.4%)
**Disease stage, n (%)**	
Active	29 (29.2%)
Remission	70 (70.7%)
**Duration of disease, n (%)**	
< 5 years	28 (28.2%)
5–10 years	22 (22.2%)
> 10 years	49 (49.4%)
**Weight, mean ± sd**	67.2 ± 13.8
**BMI**	23.02 ± 3.5
**Pharmacological therapy on-going, n (%)**	
Biologic therapies	44 (44.4%)
Adalimumab	9 (9%)
Vedolizumab	11 (11%)
Ustekinumab	8 (8%)
Infliximab	16 (16%)
Steroids	3 (3%)
Mesalazine	26 (25%)
Azathioprine	17 (17%)

### Sarcopenia and malnutrition

According to the SARC-F questionnaire, 13 subjects were sarcopenic (13.1%), with a mean score of 1.4 ± 1.7. Among the participants, 25 were malnourished: 16 satisfied the phenotypic criteria showing a BMI < 20 (mean BMI of malnourished subjects with low BMI: 18.2 ± 1.2 kg/m^2^), and nine reported weight loss greater than the 5% in the past 6 months (mean weight loss 8.1 ± 5.8 kg).

### Depression, anxiety, sleep disorders and quality of life

After completing the HADS scale, 21 (21.2%) subjects had borderline abnormal anxiety scores and 32 (32.3%) abnormal anxiety scores, with an overall mean score of 7.9 ± 4.4. Twenty subjects had borderline abnormal depression scores and six abnormal depression scores, with a mean score of 4.9 ± 3.3. A condition of altered sleep quality was found in 62 subjects (62.6%, mean score 6.9 ± 6.6). Because of the absence of established cut-offs, scores from IBDQ-SF were divided into tertiles obtaining 35 (35.3%) subjects in the first tertile (worst quality of life, score 10–42), 37 (37.3%) subjects in the second (score 43–56), and 27 (27.2%) in the third (best quality of life, score 57–70) tertile. The mean IBDQ score value was 47.8 ± 13.6.

### Fatigue: prevalence, severity and risk factors

The overall prevalence of fatigue in the sample was 92.9%, out of whom seven (7.1%) patients declared no fatigue, sixty-six (66.6%) had mild-to-moderate fatigue, and 26 (26.2%) had severe fatigue (Table 2).

**Table 2:** Univariate and multivariate analysis aimed to evaluated the risk factors associated to severe fatigue.

Variable	Subgroups	All patients	N (%) of patients with severe fatigue	Unadjusted OR (95%CI)	p value	Adjusted OR (95% CI)	p value
Age	< 40	50	14 (28)	1	0.69		
	≥ 40	49	12 (24.4)	0.83 (0.34–2.0)			
Sex	Male	57	17 (29.8)	1	0.35		
	Female	42	9 (21.4)	0.64 (0.25–1.6)			
BMI	< 25	72	21 (29.7)	1	0.28		
	≥ 25	27	5 (18.5)	0.55 (0.18–1.65)			
Type of Disease	CD	54	17 (31.4)	1	0.19		
	UC	45	9 (20)	0.54 (0.21–1.37)			
Duration of disease	< 5 years	28	8 (28.5)	1	0.91		
	5–10 years	22	6 (27.2)	0.93 (0.26–3.25)			
	> 10 years	49	12 (24.5)	0.81 (0.28–2.31)			
Biologic therapies	No	55	16 (29)	1	0.23		
	Yes	44	10 (22)	0.6 (0.2–1.43)			
Steroid therapies	No	96	25 (26)	1	0.77		
	Yes	3	1 (33)	1.4 (0.1–16.3)			
Anxiety	Absent	46	6 (13)	1	0.7	1	0.9
	Mild/moderate	21	4 (19)	1.3 (0.3–5.1)	0.001	0.9 (0.1–6.7)	0.06
	Severe	32	16 (50)	6.17 (2–18.6)		3.7 (0.9–15)	
Depression	Absent	73	13 (17.8)	1			
	Mild/moderate	20	10 (50)	4.6 (1.5–13.3)	0.004		
	Severe	6	3 (50)	6.9 (1–45.7)	0.5		
Sleep	Normal	37	4 (10.8)	1	0.002		
	Altered	62	22 (35.4)	5.2 (1.7–15.5)			
Malnutrition	No	74	15 (20.3)	1	0.02		
	Yes	25	11 (44)	3.09 (1.1–8.1)			
IBDQ score	Better Qol (II and III tertiles)	64	9 (14)*	1	< 0.001		
	Worst Qol (I tertile)	35	17 (48)	6.22 (2.3–16.4)			
Sarcopenia	No	86	17 (19.7)	1	0.001	1	0.05
	Yes	13	9 (69.2)	9.1 (2.5–33.2)		4.8 (1.1–20.1)	
Fecal Calprotectin (≥ 250 ug/dl)	Normal	70	15 (21.4)	1	0.09		
	Pathologic	29	11 (37.9)	2.4 (0.9–5.7)			
CRP (> 0,1 mg/dL)	Normal	83	18 (21.7)	1	0.02	1	0.01
	Pathologic	16	8 (50)	3.6 (1.18–10.9)		5.1 (1.3 – 20.2)	

*We merged II and III tertile because only 1 person in the III tertile (best Qol) had severe fatigue.

Based on univariate analysis, we observed that patients with severe anxiety, borderline abnormal or abnormal depression scores, low QoL, sleep disturbances, sarcopenia, malnutrition, and abnormal CRP (> 0.1 mg/dL) were more likely to report severe fatigue (Table 2). In contrast, age, type of disease, sex, disease duration, medical therapies and fecal calprotectin were not associated with fatigue (Table 2). After multivariate regression, including all significant risk factors in univariate analysis, only abnormal CRP values (OR 5.1, 95% CI 1.3–20.2), severe anxiety (OR 3.7 95% CI 0.93–15) and sarcopenia (OR 4.4, 95% CI 1.1–20.1) were identified as risk factors for severe fatigue in IBD patients.

## Discussion

Fatigue is a symptom characterized by an overwhelming sense of tiredness unresponsive to rest or sleep and causing frustration, absenteeism from work, reduction of activities and impairment of social life^13^. In subjects with IBD, fatigue is more prevalent than in a healthy population^11^, and is reported to be one of the worst symptoms during disease remission, determining higher levels of intestinal disease worries and concerns^38^. Evidences on its cause, prevalence and management are still uncertain^39^. Thus, this study was performed in order to increase our understanding on the prevalence of fatigue in patients with IBD and, at the same time, explore factors that might be associated with it. In particular, we evaluated whether muscle mass reduction could play a role in determining fatigue, in addition to the previously mentioned features. The results of our analysis show a high prevalence of fatigue in patients affected by IBD, represented by a sample composed mainly of young people with a long history of disease. We did not find any influence of sex, age, or disease duration on fatigue, whereas we found that fatigue was associated with altered CRP, severe anxiety and sarcopenia.

Our study population was mainly composed by young adults (mean age 38.7 ± 13.9 years). Nevertheless, despite their young age, the rate of subjects having sarcopenia was relevant. Sarcopenia is considered a relevant risk factor for functional age-related negative outcomes, including frailty and disability. Consistently, the parallel clinical evolution of sarcopenia and fatigue in such a young sample of patients with IBD may have important clinical implication since could represent an early alarm, in the perspective of future aging trajectories^40^.

Patients affected by IBD are subjected to a major risk of malabsorption^7^. This happens because the intestinal mucosa is not fully functional, especially when the disease is more active, because of the possible presence of complications such as fistulae, loss of mucosal absorptive surface, bowel resections, and because of the pharmacological therapies such as corticosteroids, which cause insulin resistance and leads to loss of calcium, vitamin D, albumin, and immunoglobulins^41^. Moreover, chronic inflammation and anorexia may increase energy expenditure and food intake^10^. In particular, sarcopenia is a condition of reduced muscle mass, strength, and function that impacts people affected by CD more than those affected by UC, with an estimated prevalence according to the European Society for Clinical Nutrition and Metabolism (ESPEN) of 28% and 13%, respectively^42^. The loss of lean mass is more prevalent, and happens at younger age, in people affected by IBD than in healthy individuals^43^. Among our patients, overall prevalence of sarcopenia was 13%, less than that reported in the literature, probably because of limitations of the SARC-F in detecting severe sarcopenia^27^. Nevertheless, its prevalence and the association observed following multivariate regression analysis suggest that sarcopenia represents a major contributor to fatigue in some patients with IBD.

In contrast to other authors^9,26,34^, we did not find a different prevalence of severe fatigue between UC and CD or among patients using different pharmacological treatments. This could be due to the fact that in our cohort only a few patients were taking corticosteroids, the therapy most often associated with fatigue in previous studies. Concerning anti-TNF therapies, adalimumab was reported have no effect on fatigue in a Cochrane meta-analysis and data about vedolizumab were too limited to perform any sub-analysis^39^. Thus, their efficacy in relieving fatigue is still questionable, and our study is in line with this interpretation^39^. Consistent to what is reported in literature, we did not observe that severe fatigue was more frequent in subjects with active IBD (as defined by fecal calprotectin), but we found a higher risk in subjects with abnormal CRP, in keeping with the observation of higher levels of inflammatory mediators in subjects experiencing fatigue and depression^44^.

The study design does not allow to evaluate the inference of a causal relation between sarcopenia and fatigue. We can only hypothesize, as proposed in the theoretical framework, that fatigue could be caused by lean mass reduction, in turn maybe caused by malnutrition^45^, a condition which affected 25% of our sample. On the other hand, it is also possible that fatigue perception causes a reduction in physical activity and therefore a reduction of muscle mass, sustained by malnutrition. This is the main hypothesis behind a study analyzing the relationship between muscle loss and systemic lupus erythematosus—related fatigue in a small sample of patients. The authors observed, via MRI, a voluntary reduced muscle contraction in subjects perceiving higher exertion, concluding toward a sarcopenia driven by fatigue, rather than the opposite^46^. The two interpretations however, instead of opposite, could be seen as a self-powered vicious cycle. This finding is consistent with the literature concerning strategies to treat fatigue: physical activity has already been identified as a valid therapeutic intervention for different cancers^47–49^ and two exploratory studies showed promising effects in subjects affected by IBD^39,50^. In the first study, investigators prescribed individual advice to increase physical activity by 30%, with some suggestion of the efficacy of this intervention^50^. In the second study the intervention was composed of three sessions of 60 min of strength exercises for 26 consecutive weeks, resulting in an improvement in quality of life and lumbar bone mineral density, and a decrease of fatigue perception measured with the IBD-F questionnaire^39^.

Fatigue resulted significantly related with severe anxiety and borderline abnormal or abnormal depression scores, confirming its connection on patient’s mental wellbeing. Subjects experiencing fatigue were more likely to have abnormal anxiety and depression scores, sleep disorders and to have lower quality of life scores (whose prevalence in our sample was of the 73%). This latter relationship has been described by others^19^, and may be linked to the burden of living with a chronic disease^38^, or even a physiological response to inflammation mediated by cytokines^30^. Sleep disturbances are recurrent in subjects affected by IBD also suffering for anxiety and depression^5^, worsening patients’ quality of life^5,51^. Poor sleep quality has already been associated with fatigue^20^, and the compresence of these two symptoms is considered an alert to deepen not only the presence of psychological disorder, but also of the gastrointestinal disease activity^51^. These two variables, however, lost significance in the multivariate analysis. Previous literature described the existence of a bidirectional link between fatigue and psychological dysfunctions, indicate as realistic both the hypothesis of fatigue as causative factor and the idea that negative feelings, maladaptive behaviors, and negative perception about the symptoms can worsen the perception of it^52^.

Our multivariate model suggests that sarcopenia, altered CRP and severe anxiety were the only independent risk factors for severe fatigue. In particular, we observed that patient with altered CRP and sarcopenia had an 5.1 and 4.4 higher odds of reporting severe fatigue respectively, while patients with severe anxiety had an 3.7 higher risk of severe fatigue. This finding is in line with the results of Patino-Hernandez et al., who found, in a study performed in an elderly population, that sarcopenia was significantly related to fatigue and depression, but when adjusted for confounding, only fatigue was independently related to it^53^. The authors explained this phenomenon with the overlap of items between depression and fatigue measurement tools. In contrast, Norden et al*.* in a study on fatigue in tumor-bearing mice, observed that the animals developed fatigue and a depressive-like behavior prior to muscle loss, in this case caused by the presence of the tumor^44^. The authors pointed out depressive symptom as the first determinant in developing fatigue, with sarcopenia and the worsening of quality of life as the main consequences.

This study has some limits, first of all the diagnosis of sarcopenia was based only on the SARC-F questionnaire. The European Consensus stated clearly about the fact that strength exercises and muscle quantity measures represent the gold standard of sarcopenia assessment, and a more accurate study should conduct these procedures. However, due to the restrictions related to the risk of COVID transmission, we reduced access to the hospital for all patients and therefore more objective assessments were not possible. Moreover, the same pandemic affected our ability to recruit patients, thus resulting in a limited sample size, together with a lower response rate (40.5%) to the survey, as compared with other studies investigating fatigue by means of questionnaire (55%). Further, the cross-sectional design of this investigation did not allow us to establish if the presence of severe fatigue caused a low sarcopenia or vice versa*.* Despite the importance of the physical activity, we did not ask the patients about this in the survey. Lastly, diagnosis of active disease was not based on objective evidence like endoscopy or radiology, but on a fecal calprotectin value, although this is widely accepted as a good biomarker of disease activity in patients with IBD^54^. Nevertheless, the study suggests a connection between fatigue and sarcopenia. What is clear is that when studying a complex phenomenon like fatigue, it cannot be done without taking into account both physical and psychological health.

In conclusion, we confirm that fatigue is a symptom with a high prevalence in subjects affected by IBD and sarcopenia, a condition caused by malnutrition and the state of chronic inflammation typical of these patients, is an important risk factor for it. Moreover, we observed a higher prevalence of severe fatigue in subjects with severe anxiety. Thus, subjects affected by IBD should be asked about the presence of fatigue during clinical evaluation, above all in case of concomitant symptoms such as anxiety, depression or bad sleep quality, and they should eventually be referred to a professional for a consultation. The role of physical activity in protecting from fatigue seems promising and should be encouraged, when considered appropriated, because of its role in preventing lean mass loss and therefore maintaining individuals’ autonomy, preventing from hospitalization and improving quality of life. Further research on the underlying causes of fatigue, as well as effective interventions to treat it, are needed.

## Data Availability

The datasets used and/or analyzed during the current study are available from the corresponding author on reasonable request.
